# Heat-Killed *Leuconostoc mesenteroides* H40 Alleviates Cognitive Impairment by Anti-Inflammation and Antioxidant Effects in a Scopolamine-Induced Mouse Model

**DOI:** 10.4014/jmb.2411.11013

**Published:** 2025-02-25

**Authors:** Na-Kyoung Lee, Yunjung Lee, Jiyoung Hwang, Eunju Park, Hyun-Dong Paik

**Affiliations:** 1 Department of Food Science and Biotechnology of Animal Resources, Konkuk University, Seoul 05029, Republic of Korea; 2 Department of Food and Nutrition, Kyungnam University, Changwon 51767, Republic of Korea; 3 View of Creativity, GHBio Co., Ltd., Konkuk University, Seoul 05029, Republic of Korea

**Keywords:** Probiotics, *Leuconostoc mesenteroides*, cognition, anti-neuroinflammatory effect, antioxidant effect

## Abstract

*Leuconostoc mesenteroides* H40 (H40), originally isolated from kimchi, has been shown to exhibit probiotic characteristics and a neuroprotective effect in SH-SY5Y cells. In this study, we investigated the modulatory effects of heat-killed H40 (H-H40) in a scopolamine-induced (1 mg/kg/day) mouse model of cognitive impairment. H-H40 at either 1 × 10^8^ or 1 × 10^9^ CFU/day alleviated scopolamine-induced cognitive impairment in the novel object recognition and Y-maze tests. Neuroinflammatory cytokines, tumor necrosis factor (TNF)-α, interleukin (IL)-1β, inducible NO synthase (iNOS), and cyclooxygenase (COX)-2 were all found to be decreased by H-H40 treatment. Moreover, changes in neurotransmitter levels and synaptic plasticity were further confirmed through measurement of acetylcholinesterase, acetylcholine, choline acetyltransferase, and brain-derived neurotrophic factor (BDNF) levels. H-H40 increased β-secretase levels, but decreased amyloid-β levels. In addition, the antioxidant effects of catalase and GPx were demonstrated. Overall, our results showed that H-H40 exerts positive cognitive effects by anti-inflammatory and antioxidant activities in a mouse model of scopolamine-induced cognitive impairment. H-H40 could be used as a prophylactic functional food for improving cognition.

## Introduction

Cognitive function is influenced by aging, environmental exposure to hazardous contaminants (such as drugs or alcohol), brain injury, and social correlations [1]. Alzheimer’s disease (AD) is a neurodegenerative disorder accompanied by both mental and physical weakening [2]. The primary symptoms of AD include cognitive decline, impaired reasoning and judgment, short-term memory loss, and communication difficulties [3]. At the molecular level, AD is characterized by the accumulation of extracellular amyloid-β (Aβ) peptides in the brain [4]. This accumulation of Aβ is associated with neuroinflammation, mitochondrial apoptosis, and synaptic dysfunction. Consequently, Aβ accumulation and oxidative stress are generally acknowledged as major factors affecting AD.

The gut–brain axis is a bidirectional network that links the enteric and central nervous systems (CNS). In addition to anatomical aspects, this network also extends to include humoral, endocrine, immune, and metabolic pathways. Psychobiotics are probiotics that deliver or produce neuroactive substances that can benefit patients with neurodegenerative diseases via the gut-brain axis. The hippocampus is the primary brain region accountable for the formation and consolidation of fresh memories, with crucial influence on adult hippocampal neurogenesis [5].

Oxidative stress and neuroinflammation is associated with neurodegenerative diseases, such as AD, amyotrophic lateral sclerosis (ALS), and Parkinson’s disease (PD) [6, 7]. Excessive oxidative stress can also lead to inflammatory conditions, such as neurodegenerative diseases [8]. Neuroinflammation is a defense mechanism that protects the brain by removing or preventing various pathogens [9]. This inflammatory stimulation can persist due to endogenous factors, such as infection, trauma, and drugs, protein aggregation, genetic mutation, or environmental factors [10].

Lactic acid bacteria have previously been reported to exhibit numerous health functions, including anticancer and neurodegenerative effects, through antioxidant and anti-inflammatory activities [11-14]. Probiotics are living organisms that provide health benefits to their hosts. *Leuconostoc mesenteroides* is a heterolactic fermentative bacterium found in the early stages of kimchi fermentation. Some *L. mesenteroides* strains have been used as probiotics [11]. In addition, extracellular vesicles from *L. mesenteroides* have demonstrated anti-inflammatory activities in microglial cells [12]. Recently, paraprobiotics, or heat-killed probiotics, have gained attention because of their novel functions and stability [13]. *L. mesenteroides* H40, a bacterial strain isolated from kimchi, has been shown to exhibit probiotic properties and neuroprotective effects in SH-SY5Y cells (neuroblastoma cell line) [14]. Heat-killed *L. mesenteroides* H40 (H-H40) has not yet been demonstrated to influence cognitive function in scopolamine-treated mouse models. Hence, we aimed in the present study to research the preventive effects of H-H40 against cognitive impairment in scopolamine-treated model mice.

## Materials and Methods

### Probiotic Samples

*L. mesenteroides* H40 cells were cultivated as probiotics in lactobacilli MRS media and centrifuged for collection [14]. Two washed *L. mesenteroides* H40 cells were resuspended in phosphate-buffered saline (PBS) (HyClone, USA) or 1% glucose. To obtain H-H40, suspended H40 cells were heated at 80°C for 30 min, and the harvested cells were then freeze-dried. These freeze-dried H-H40 cells were then used in an animal model.

### Mice and Experimental Design

All animal protocols performed in this study followed the guidelines of the Institutional Animal Care Committee of Kyungnam University (KUICA-22-02). ICR mice (7-weeks-old, male) were acquired from Koatech (Republic of Korea). Mice were contained in groups of three to four per cage at a constant temperature of 23 ± 2°C and relative humidity of 53 ± 2%, with a 12-h light/dark cycle. After one week of adaptation, the mice were divided into five groups (*n* = 10 per group), as follows: normal control (NC, no treatment), positive control (PC, scopolamine-injected control, 1 mg/kg/day), H-H40L (treated with scopolamine 1 mg/kg/day, and 1 × 10^8^ CFU/day H-H40), H-H40H (treated with scopolamine 1 mg/kg/day, and 1 × 10^9^ CFU/day H-H40), and DO (treated with scopolamine and donepezil, 2 mg/kg; medicinal control). The samples or donepezil were administered for 2 weeks, and scopolamine was injected intraperitoneally for 7 to 14 days to induce memory impairment starting one week before the experiment.

The animals were starved for 12 h and then sacrificed with 4 ml/kg isoflurane. Blood samples were gathered and centrifuged (2,000 ×*g* for 30 min) to split the serum. Each organ was separated on ice and frozen at –80°C for further study.

### Behavioral Tests

To conduct the behavioral tests, we used novel object recognition and Y-maze tests. For the novel object recognition test, mice were set in a box plus two similar objects (O1+O2), and permitted to adapt for 3 min. The consumed time with each object and touched number over 5 min were documented. The next day, object O1 was switched with object O2. The mice were allowed to explore the objects for 5 min, and the time consumed on the new object was used to estimate the recognition index. The touched numbers on a new object represents the cognitive recognition of that object.

The Y-maze test was performed following the method of Salahuddin *et al*. [15], with some modifications. A device with three arms (50 cm (length) × 20 cm (height) × 10 cm (width)) was used. The arms were tapered 10 cm away from the free ends. All mice experienced two training trials per day over two serial days. Each mouse was set at the end of one arm and admitted to move freely through the maze for 8 min. During the trial assemblies, the mice were set at the center of the apparatus and allowed to move generously for 8 min to explore the arms at random. The experimental assemblies were conducted over 4 days. The total number of entries into each arm was recorded during each session. The spontaneous alternation behavior of the mice was calculated using the following formula:

$$\text{Spontaneous alternation (\%)}=\frac{\text{Number of alternations}}{\text{Total number of arm entries}-2}\times 100$$

### Neuroinflammatory Cytokines, iNOS, and COX-2

Hippocampal tissues were standardized and these cells were then centrifuged (10,000 ×*g*, 5 min) to obtain supernatants. TNF-α and IL-1β were analyzed using a commercial enzyme-linked immunosorbent assay (ELISA) kit (BD, USA). The absorbances of TNF-α and IL-1β were measured at 450 nm using a microplate reader.

Total RNA was extracted from the brain tissue using TRIzol reagent (Invitrogen, USA), and cDNA was synthesized from 1 μg of RNA using M-MLV reverse transcriptase (Promega, USA). After cDNA synthesis, quantitative real-time PCR was performed using 25 μl of SYBR Green master mix (PhileKorea, QuantiSpeed SYBR No-ROX Kit, Republic of Korea ) with a real-time DNA thermal cycler (CFX Duet Real-Time PCR System, Bio-RadUSA). The reaction mixtures were finally incubated for an initial denaturation at 95°C for 10 min, followed by 50 cycles of PCR. Each cycle was performed as per the following parameters: 95°C for 10 s, 55°C for 20 s, and 72°C for 20 s. The sequences of the sense and antisense primers used for amplification are listed as follows: *COX-2*, sense, 5'-acctggtgaactacgactgc-3' and antisense, 5'-ctagggaggggactgctcat-3'; *iNOS*, sense, 5'-ctatggccgctttgatgtgc-3' and antisense, 5'-tggggatgctccatggtcac-3'. The β-actin-encoding gene was used as a reference gene. Normalized target gene expression levels in each sample were calculated using 2^-ΔΔCT^. Values are expressed as the fold change compared to control, and are presented as the mean ± SE (*n* = 7).

### β-Secretase and Amyloid-β Contents

The hippocampal tissues were standardized and these cells were then centrifuged (10,000 ×*g*, 5 min) to harvest the supernatants. The β-secretase levels in supernatants were measured using the Mouse Bace1 ELISA Kit (Express Biotech International, USA).

To obtain Aβ1-40 and Aβ1-42 levels, the hippocampal tissue was homogenized in 5 M guanidine-HCl/50 mM Tris buffer (8×, pH 8.0) on ice. This hippocampal homogenate was incubated at room temperature for 3–4 h, mixed with cold BSAT-PBS (10×), and centrifuged (16,000 ×*g*, 20 min) to acquire the supernatant. Aβ1-40 and Aβ1-42 levels in the supernatant were determined using the Mouse Amyloid β40 and β42 ELISA kits from BioVendor R&D (Czech Republic), respectively.

### Acetylcholine, Acetylcholinesterase, and Choline Acetyltransferase

Hippocampal tissues were standardized and centrifuged (10,000 ×*g*, 5 min) to get the supernatant. Obtained supernatants were measured to analyze the AChE, ACh, and choline acetyltransferase (ChAT) activities. These ELISA kits were obtained from Bioassay Systems (USA) and MyBiosource, Inc. (USA).

### *BDNF* Level

*BDNF* levels were investigated in the hippocampus tissue. The rt-PCR was conducted similarly to cytokine expression. The sequences of the sense and antisense primers used for amplification are listed in Lee *et al*. [14].

### Antioxidant Activity

Hippocampal tissues were standardized and centrifuged (10,000 ×*g*, 5 min) to get a supernatant. Then, the catalase and glutathione peroxidase (GSH-Px) activities were measured. Catalase activity was calculated following the method of Ostovan *et al*. [16], with some modifications. First, 100 μl of supernatant was dissolved in 50 ml of 50 mM phosphate buffer (pH 7), and 2 ml of the mixture was added to a cuvette. The reaction was then initiated through the addition of 1 ml of 30 nM H_2_O_2_ at 20°C. The H_2_O_2_ decomposition rate was measured at 240 nm for 30 s using a spectrophotometer.

GSH-Px levels were determined according the method of Martens *et al*. [17]. First, 10 μl of supernatant was added to 100 μl of 1 M Tris-HCl-5 mM EDTA buffer (pH 8.0), 100 μl of 10 U/ml glutathione reductase, 20 μl of 0.1 M glutathione, and 100 μl of 2 mM NADPH, and filled with distilled water to 1 ml. After incubation at 37°C for 10 min, the reaction was begun by the addition of 10 μl of t-butyl hydroperoxide, and the absorbance was observed at 340 nm. The reaction was run for 90 s, and NADPH loss was monitored based on the change in the A_340_/min.

### Statistical Analyses

All data were analyzed using one-way analysis of variance (ANOVA) and Duncan’s multiple range test for multiple comparisons. SPSS software (IBM Corp., USA) was used for statistical analyses and the results were considered statistically significant on the basis of *p* < 0.05.

## Results

### Effect of H-H40 on Behavioral Tests

**Fig. 1:**
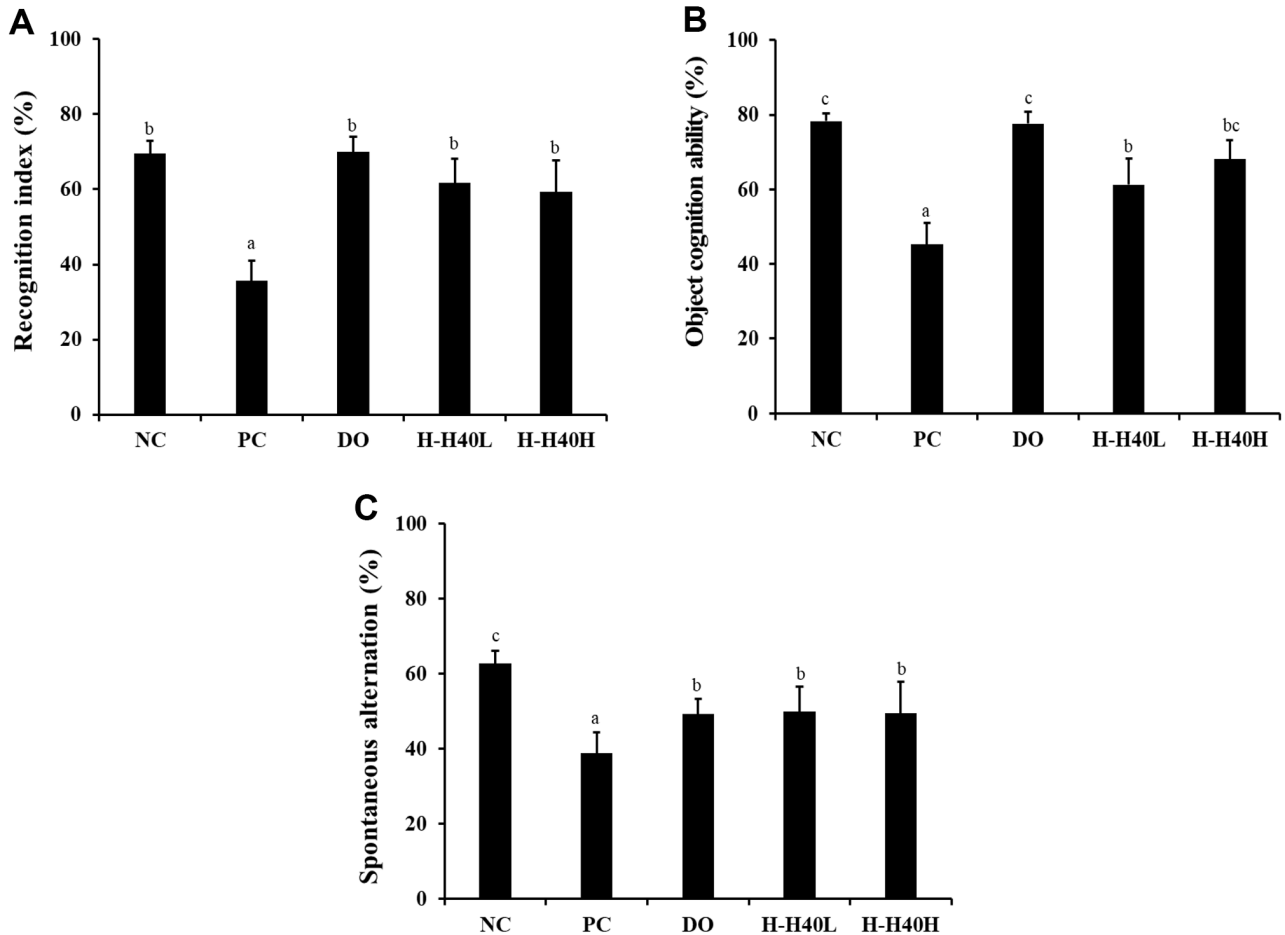
Behavioral effects of heat-killed *Leuconostoc mesenteroides* H40 using the novel object exploration and Y-maze test in mice with scopolamine-induced memory impairment (*n* = 10). (**A**) Recognition index (%) (**B**) object cognition ability (%), and (**C**) spontaneous alternation (%). NC, normal control without scopolamine; PC, positive control with scopolamine; DO, administration of 2 mg/kg donepezil with scopolamine; H-H40-L, administration of 1 × 10^8^ CFU/day H-H40 with scopolamine; H-H40-H, administration of 1 × 10^9^ CFU/day H-H40 with scopolamine. Data are presented as the mean ± standard deviation, and alphabetic letters on the error bars indicate significant differences (*p* < 0.05).

The recognition index and object cognitive ability were investigated using new objects to reveal changes in scopolamine-treated mice. Specifically, the scopolamine-treated control (PC) group (35.6%) represented a significant reduction in the recognition index over time for new objects compared to the NC group (69.5%)(Fig. 1A). The H-H40-L and H-H40-H groups revealed 61.8% and 59.3%, respectively. These values indicated a significant increase in contrast with the PC group. With regard to object cognitive ability, the PC group (45.3%) had a significantly decreased cognition ability as the touched number of new objects increased, compared to the NC group (78.3%) (Fig. 1B). The H-H40-L and H-H40-H groups showed 61.3% and 68.1%, respectively. A slight increase showed contrast with the PC group. The Y-maze test exposed evident patterns of spontaneous alternations in the tested groups (Fig. 1C). The PC group (38.9%) showed significantly lowered alternation behavior compared with the NC group (62.7%), indicating the induction of cognitive impairment. Furthermore, all H-H40 or DO groups showed a significant increase in spontaneous alternation compared to the PC group, thus demonstrating the effect of improved cognitive ability. Therefore, in all behavior tests, the H-H40 group showed similar results to that in the DO group, with no significant differences between groups.

### Anti-Neuroinflammatory Effect of H-H40 in Hippocampal Protein

**Fig. 2:**
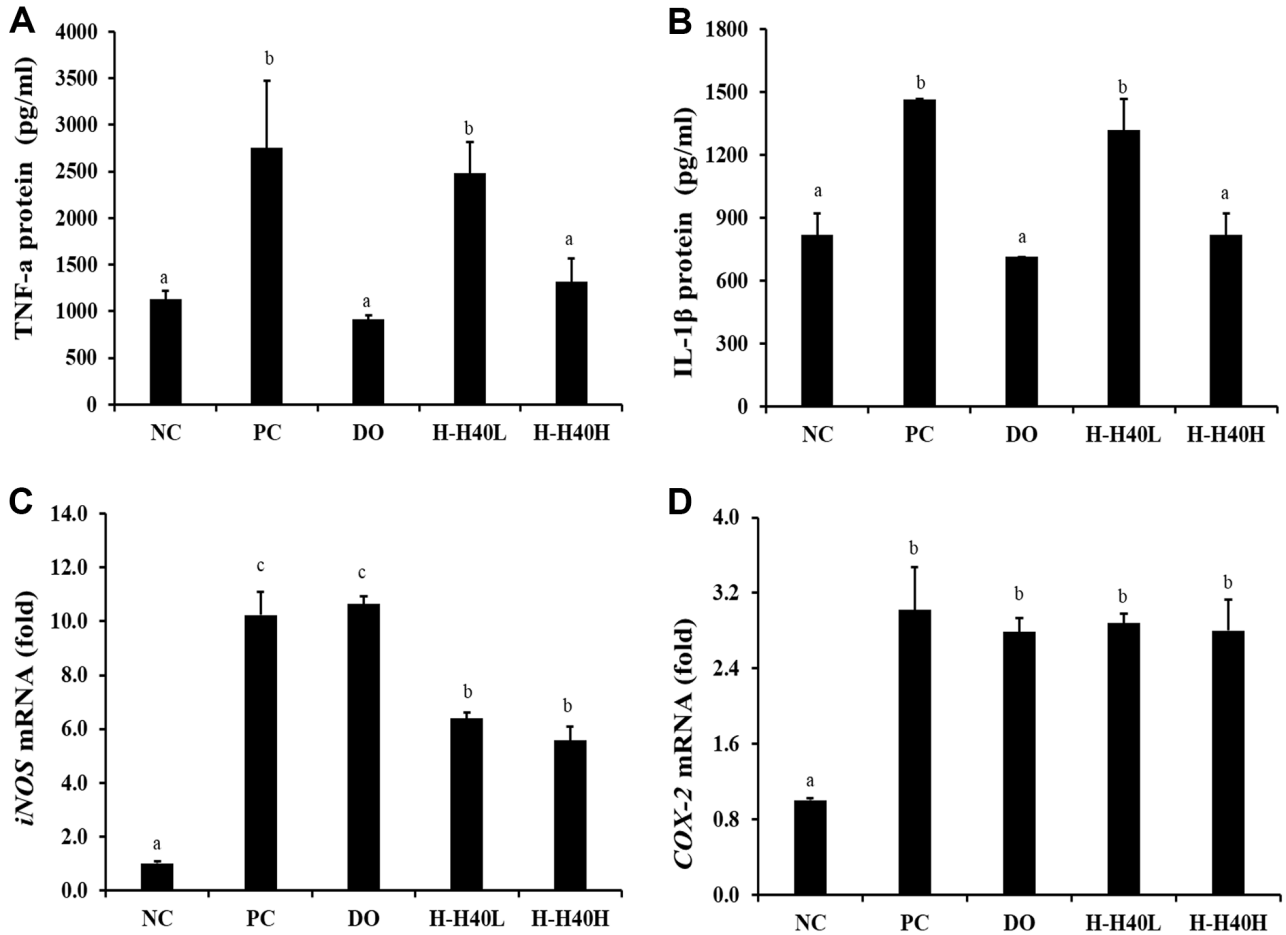
Anti-neuroinflammatory effect of heat-killed *L. mesenteroides* H40 on hippocampal proteins. Protein expression of (**A**) TNF-α and (**B**) IL-1β. mRNA expression of (**C**) iNOS and (**D**) COX-2. NC, normal control without scopolamine; PC, positive control with scopolamine; DO, administration of 2 mg/kg donepezil with scopolamine; H-H40-L, administration of 1 × 10^8^ CFU/day H-H40 with scopolamine; H-H40-H, administration of 1 × 10^9^ CFU/day H-H40 with scopolamine. Data are presented as the mean ± standard deviation, and alphabetic letters on the error bars indicate significant differences (*p* < 0.05).

TNF-α and IL-1β protein production levels were assessed (Fig. 2A and 2B). The PC group (2,756.5 pg/ml) showed increased TNF-α production compared to the NC group (1,134.5 pg/ml), while the values in the H-H40-H and DO groups were 1,314.7 and 917.1 pg/ml, respectively. The PC group (1,466.2 pg/ml) also showed an increase in IL-1β production compared to the NC group (818.8 pg/ml). The corresponding values in the H-H40-H and DO groups were 818.8 and 714.4 pg/ml, respectively. Notably, the H-H40 groups exhibited concentration-dependent results.

The mRNA expression levels of iNOS and COX-2 were also determined (Fig. 2C and 2D). Specifically, the PC group (10.2-fold) showed a significant increase in iNOS in brain tissue compared to the NC group (1.0-fold), indicating damage to brain tissue cells following scopolamine treatment. However, the H-H40-L and H-H40-H groups showed 6.39- and 5.58-fold lower expression levels, respectively. Although the DO group showed a higher expression value (10.64-fold), these values were similar to those observed in the PC group. For COX-2, the PC group (3.02-fold) showed an increased value compared to the NC group (1.00-fold). Groups H-H40-L, H-H40-H, and DO further exhibited slightly lower expression levels (2.88-, 2.80-, and 2.78-fold, respectively).

### Effect of H-H40 on Amyloid-β Accumulation in Brain Tissue

**Fig. 3:**
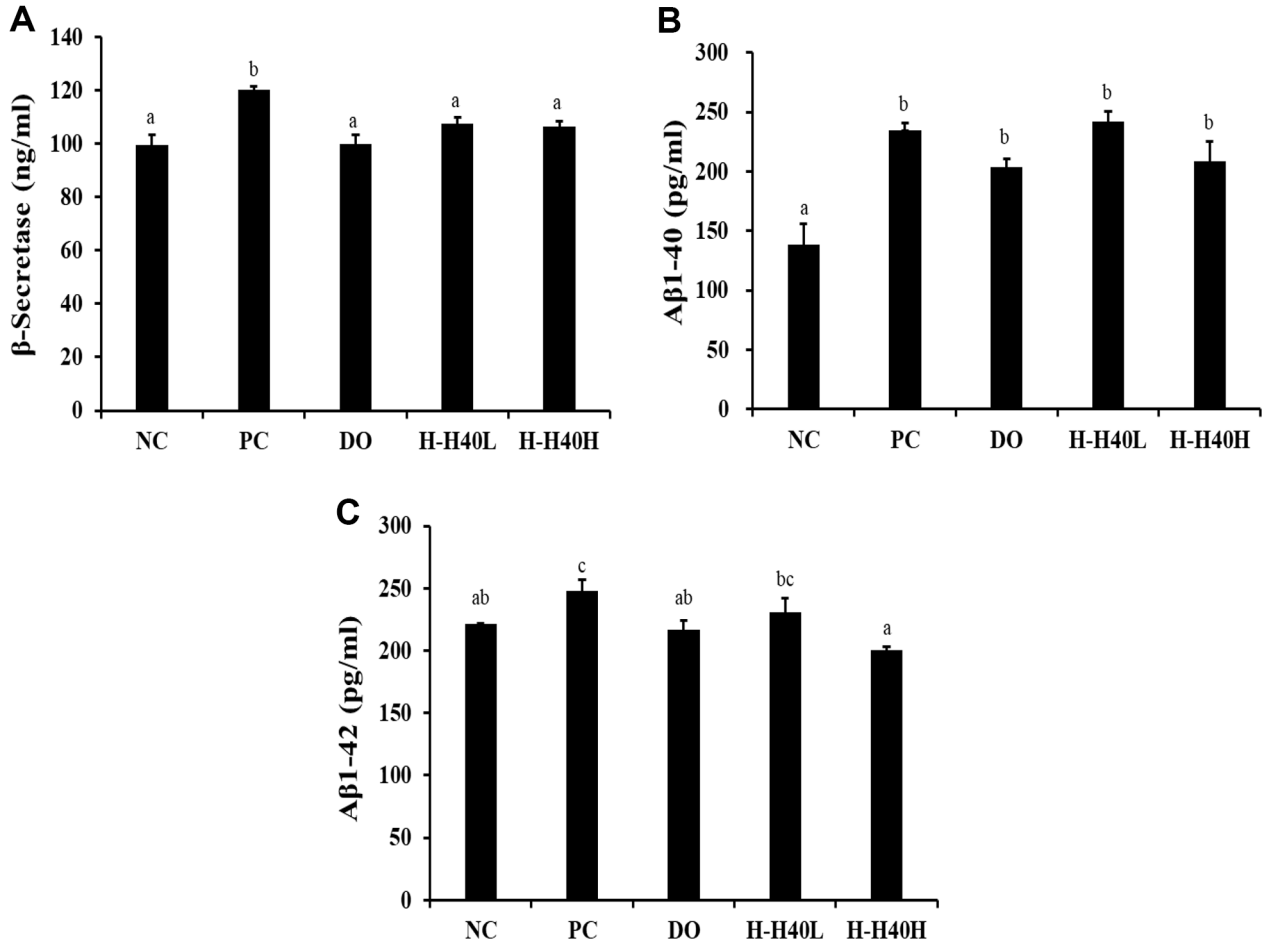
Effects of heat-killed *L. mesenteroides* H40 on amyloid β accumulation in the brain tissue, as assessed by ELISA. (**A**) β-Secretase, (**B**) amyloid β1-40 (Aβ1-40), and (**C**) amyloid β1-42 (Aβ1-42) activities. NC, Normal control without scopolamine; PC, positive control with scopolamine; DO, administration of 2 mg/kg donepezil with scopolamine; HH40- L, administration of 1 × 10^8^ CFU/day H-H40 with scopolamine; H-H40-H, administration of 1 × 10^9^ CFU/day H-H40 with scopolamine. Data are presented as the mean ± standard deviation, and alphabetic letters on error bars indicate significant differences (*p* < 0.05).

The PC group (120.2 ng/ml) displayed significant increase of β-secretase levels compared with the NC group (99.4 ng/ml) (Fig. 3A). All H-H-40 and DO groups (49.3–50.0 ng/ml) represented a significant decrease of β-secretase levels contrasted with the PC group.

The Aβ1-40 content showed significant increase in the PC group (234.9 pg/ml) compared with the NC group (138.3 pg/ml) (Fig. 3B), while the corresponding values in the H-H40-H and DO groups were 208.3 pg/ml and 203.3 pg/ml, respectively. In addition, the Aβ1-42 content was found to be increased in the PC group (247.9 pg/ml) compared with the NC group (221.5 pg/ml) (Fig. 3C), with values of 200.3 pg/ml and 216.7 pg/ml in the H-H40-H and DO groups, respectively. These results showed a slight decrease in Aβ accumulation.

### Effect of H-H40 on Neurotransmitters

**Fig. 4:**
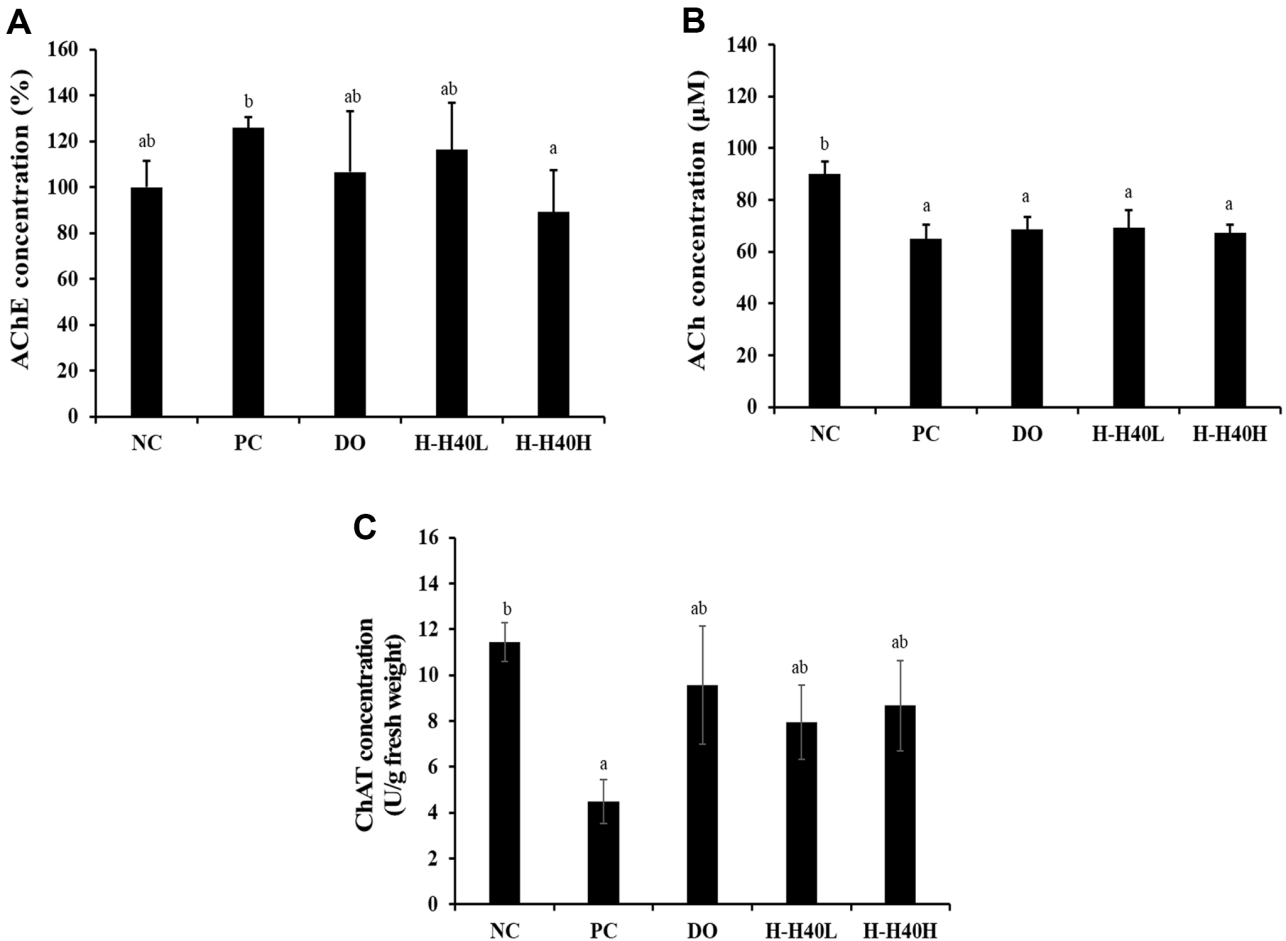
Effects of heat-killed *L. mesenteroides* H40 on choline-based substances in brain tissue, assessed by ELISA. Production of (**A**) acetylcholinesterase (AChE), (**B**) acetylcholine (ACh), and (**C**) choline acetyltransferase (ChAT). NC, Normal control without scopolamine; PC, positive control with scopolamine; DO, administration of 2 mg/kg donepezil with scopolamine; H-H40-L, administration of 1 × 10^8^ CFU/day H-H40 with scopolamine; H-H40-H, administration of 1 × 10^9^ CFU/day H-H40 with scopolamine. Data are presented as the mean ± standard deviation, and alphabetic letters on the error bars indicate significant differences (*p* < 0.05).

Fig. 4A shows that the PC group (125.9%) exhibited significant increase of AChE activity compared to the NC group (100%). The H-H40-L (116.6%), H-H40-H (89.2%), and DO (106.6%) groups also displayed significantly lower AChE activity than the PC group, demonstrating enhanced cognitive ability.

The ACh content was reduced in the PC group (64.91 μM) compared with that in the NC group (90.06 μM)(Fig. 4B). The H-H40-L (69.28 μM), H-H40-H (67.33 μM), and DO (68.91 μM) groups all showed slight increases compared to the PC group. However, this difference was not statistically significant.

In the ChAT activity (Fig. 4C), the PC group (4.48 U/g fresh weight) showed significant decrease of ChAT activity compared with the NC group (11.44 U/g fresh weight), while the H-H40-L (7.94 U/g fresh weight), H-H40-H (8.66 U/g fresh weight), and DO (9.56 U/g fresh weight) groups displayed significant increase of ChAT activity compared to the PC group.

### Effect of H-H40 on Synaptic Plasticity

**Fig. 5:**
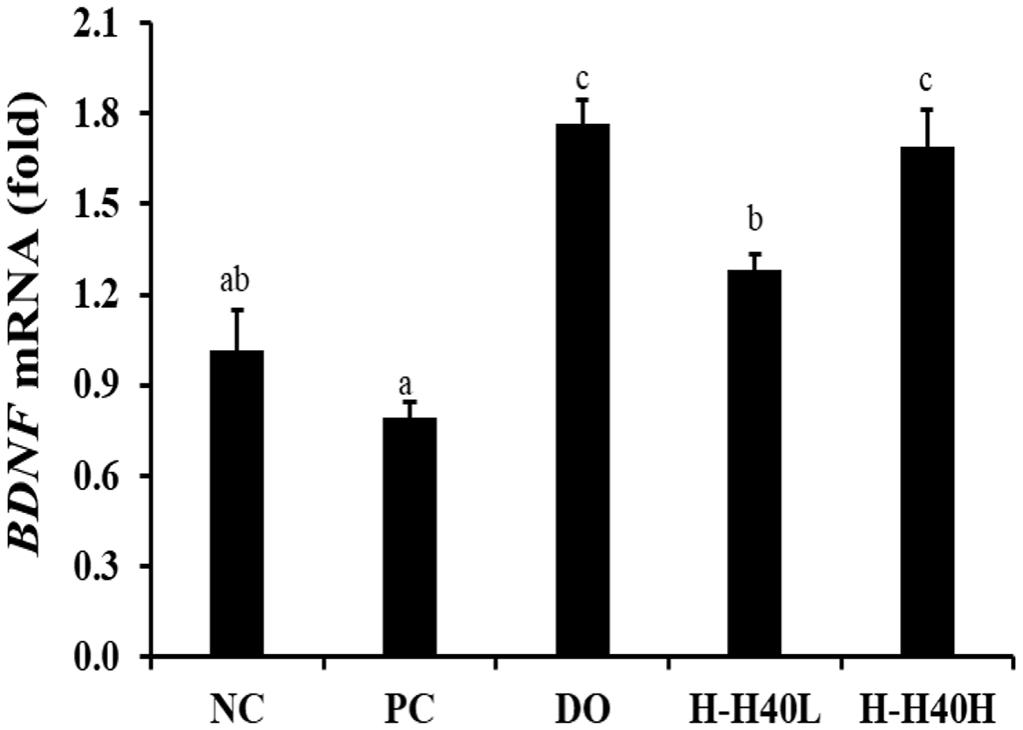
Effects of heat-killed *L. mesenteroides* H40 on *BDNF* mRNA expression. BDNF, brain-derived neurotrophic factor; NC, normal control without scopolamine; PC, positive control with scopolamine; DO, administration of 2 mg/kg donepezil with scopolamine; H-H40-L, administration of 1 × 10^8^ CFU/day H-H40 with scopolamine; H-H40-H, administration of 1 × 10^9^ CFU/day H-H40 with scopolamine. Data are presented as the mean ± standard deviation of triplicate experiments. Different letters on the error bars indicate significant differences (*p* < 0.05).

In Fig. 5, the *BDNF* mRNA expression levels showed the variation in the hippocampi of mice (Fig. 5). The PC group (0.79-fold) displayed significant decrease of *BDNF* expression compared with the NC group (1.02-fold), while the H-H40-L (1.28-fold), H-H40-H (1.69-fold), and DO group (1.77-fold) showed significant increases in *BDNF* expression compared with the PC group, representing an enhancement in cognitive ability.

### Antioxidant Effect of H-H40 in Hippocampal Protein

**Fig. 6:**
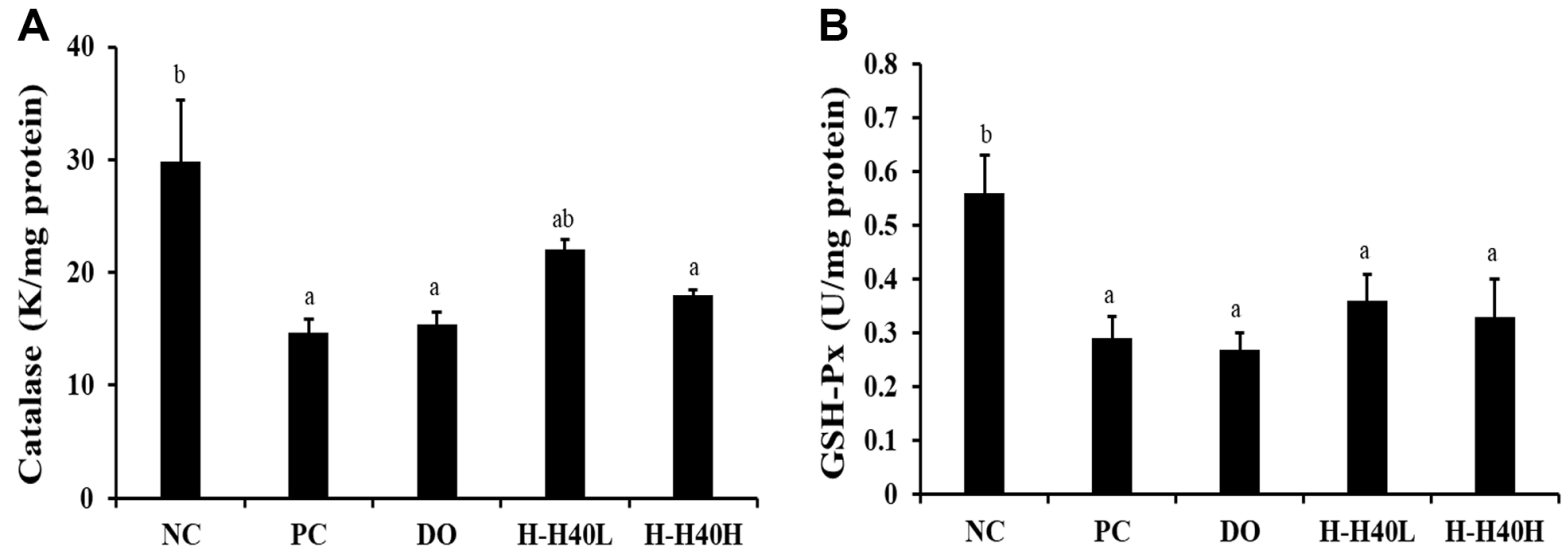
Antioxidant effects of heat-killed *L. mesenteroides* H40. Activity of (**A**) catalase and (**B**) GSH-Px. NC, normal control without scopolamine; PC, positive control with scopolamine; DO, administration of 2 mg/kg donepezil with scopolamine; H-H40-L, administration of 1 × 10^8^ CFU/day H-H40 with scopolamine; H-H40-H, administration of 1 × 10^9^ CFU/day H-H40 with scopolamine. Data are presented as the mean ± standard deviation and alphabetical letters on the error bars indicate significant differences (*p* < 0.05).

The antioxidant effects of H-H40 were confirmed by catalase and GSH-Px production. In terms of catalase poduction, the PC group (14.7 K/mg protein) showed a significant decrease in the brain tissue compared with the NC group (29.8 K/mg protein), while brain tissue cells were oxidized by scopolamine treatment (Fig. 6A). H-H40 groups (18.0–22.0 K/mg protein) showed lower catalase activity, while the DO group showed the lowest value, at 15.4 K/mg protein.

In regard to GSH-Px, the PC group (0.29 U/mg protein) showed a significant decrease in the brain tissue compared with the NC group (0.56 U/mg protein), while brain tissue cells showed increased oxidation following scopolamine treatment (Fig. 6B). H-H40 groups (0.33–0.36 U/mg protein) showed relatively high GSH-Px activity, while the DO group showed the lowest value, at 0.27 U/mg protein.

## Discussion

Overall, in the present study, we investigated the cognitive-alleviating effects of H-H40 in a mouse model of scopolamine-induced cognitive impairment. H-H40 treatment further exhibited improved learning and memory capacities, and suppressed neuroinflammation and Aβ accumulation. Furthermore, H-H40 treatment increased the neurotransmitter and antioxidant effects.

Scopolamine, aging, and LPS have all been used in mouse models of cognitive impairment [6, 18]. Among these conditions, scopolamine has been shown to cause deficits in learning acquisition and consolidation, in addition to promoting neuroinflammation and oxidative stress [19]. Cholinergic neurons in the CNS degenerate in a manner that associates with functional loss of cognition in patients with AD and senile dementia. Fig. 1 shows the cognitive variations induced by scopolamine treatment. The PC group treated with scopolamine exhibited reduced cognitive function and spontaneous alternation, while treatment with H-H40 or donepezil alleviated the effects of scopolamine. These behavioral benefits could be confirmed by administration of H-H40. However, to explain the mechanisms of the neuroprotective effects of H-H40, additional research, including on the intestinal microbiome, appears to be necessary.

Neuroinflammation is activated by the conjugation of immune cells in the CNS connecting microglia and astrocytes. This neuroinflammation can accelerate neurological deficits, whereas inhibition of the inflammatory responses can be neuroprotective [20]. IL-1β and TNF-α are directly related to brain function and can promote inflammatory response inside the brain [21, 22]. IL-1β is associated with the inflammatory activity, including the accumulation of Aβ peptides, and has further been linked to stroke, AD, and multiple sclerosis [22]. Increased TNF-α has also been shown to be associated with lower hippocampal volume and cognitive impairment [21]. iNOS and COX-2 are neurotoxic mediators modulated by the NF-κB signaling pathway [22]. Therefore, these pro-inflammatory cytokines and mediators are also associated with brain diseases. NF-κB is an inducible regulator of inflammation in the brain. *Lactobacillus acidophilus* has shown neuroprotective effects by lowering IL-1β [22]. Folic acid ameliorated memory impairment by antioxidant effect [23]. *Levilactobacillus brevis* KU15147 also demonstrated anti-inflammatory effect in macrophage cells through action on the NF-κB, AP-1, and MAPK signaling pathways [24]. TNF-α further induces iNOS and COX-2 transcription. As shown in Fig. 2, H-H40 reduced IL-1β, TNF-α, iNOS, and COX-2 levels in a concentration-dependent manner, indicating a protective effect against neuroinflammation.

Aβ is an important initiator of AD pathology. The production of Aβ is related with mitochondria, the major source of energy for the brain. β-Secretase levels have been shown to be upregulated in dementia [25]. β-Secretase regulates ordinally amyloid precursor protein, Aβ, and Aβ plaques [26]. The inhibition of β-secretase can prevent Aβ aggregation. In our study, we found that H-H40 can lower β-secretase and Aβ production in mice treated with scopolamine, in a manner similar to treatment with donepezil (Fig. 3). Similarly, one prior study showed that *Bifidobacterium breve* MCC1274 reduced Aβ production in APP knock-in mouse by exerting anti-inflammatory effects through the modulation of the ERK/HIF-1α signaling pathway [27]. Similarly, another study showed that *Lactococcus lactis* KC24 inhibited β-secretase and Aβ production [28].

Synaptic plasticity is associated with AChE, ACh, and ChAT activities. In our study, scopolamine treatment reduced AChE, ACh, and ChAT activities (Fig. 4). ACh plays a crucial role in the peripheral and CNS, while AChE hydrolyzes ACh at the synaptic cleft. ChAT is responsible for acetylcholine synthesis, and is the most important indicator of the functional state of cholinergic neurons. Cholinergic neurons are located in the basal forebrain, including those that form the nucleus basalis of Meynert cells in AD [29]. H-H40 and donepezil treatment increased choline-related enzyme activity, with ChAT exhibiting the greatest influence. Especially, cognitive and plasticity-related factors were affected by lower doses of H-H40 than by donepezil.

Neurotransmitters include ACh, 3,4-dihydroxy-phenylacetic acid, dopamine, and homovanillic acid [30]. BDNF is related to neural plasticity and neuron survival in the release of mBDNF and pro-BDNF in cellular membrane [31]. In this study, BDNF levels were found to be reduced in the brain tissue by scopolamine treatment, and these results were comparative to memory deficits. BDNF levels have further been reported to be decreased by oxidative stress [32]. H-H40 treatment alleviated the decrease in BDNF levels (Fig. 5). Lab4b and *L. lactis* KC24 increased BDNF mRNA expression [28]. H-H40 treatment alleviated the decrease in BDNF levels (Fig. 5). Lab4b and *L. lactis* KC24 increased BDNF mRNA expression.

*Lactobacillus gasseri* NK109, *Lactiplantibacillus plantarum* MWFLp-182, *Lactobacillus johnsonii* CJLJ103, and *L. lactis* KC24 alleviated LPS, D-galactose, or scopolamine-induced cognitive impairment by upregulation of inflammation-mediated BDNF expression [28, 33-35]. *L. plantarum* MWFLp-182 further induces memory impairment and inflammation triggered by D-galactose in an aging mouse model [35]. H-KC24 and H-H40 demonstrate neuroprotective effects against oxidative stress on the apoptotic factors, *Bax* and *Bcl2* [14, 28]. *L. brevis* KU15151 has further been shown to inhibit inflammation in RAW 264.7 macrophages [36]. Together, these results confirmed the hypothesis that H-H40 alleviates cognition impairment through the modulation of neuroinflammation, neurotransmitters, synaptic plasticity, Aβ accumulation, and antioxidant effects.

## Conclusion

H-H40 treatment alleviated cognitive damage in a scopolamine-induced mouse model with cognitive impairment. Behavioral deficits were further found to be alleviated by H-H40 treatment. The effects of H-H40 were demonstrated by the control of neuroinflammation, neurotransmitters, synaptic plasticity, Aβ accumulation, and antioxidant effect in brain tissue. Although our results suggest that *L. mesenteroides* H40 is a potent, natural neuroprotective agent, for H-H40 to be employed as a preventive functional food, additional clinical research will need to be conducted.
